# Economy in the Isolation Hospital

**Published:** 1916-08-12

**Authors:** 


### ECONOMY IN THE ISOLATION HOSPITAL.

### How It May Be Secured.

The special difficulties of maintaining economy in this class of institution lie in the varying numbers of the staff and patients. Unless the numbers be carefully set forth in the yearly report, grave discontent with the household management may quite unfairly be aroused in the weekly board, and subscribers may nurse a grievance which, as it does not come into the open to be dispersed by investigation, may greatly damage the financial position.

### The Institutional Day-Book.

The first essential is to keep a day-book, showing the numbers present at the mid-day dinner. There are often out-workers in institutions, and these, if provided with dinner, should be entered as half ($\frac{1}{2}$), for the mid-day dinner stands for fully half a day's board. The book should be ruled for thirteen weeks, and should give particulars concerning the following: Medical and secretarial staff, nursing staff, visitors, domestic staff, and patients under the different wards or all together, as preferred. It would be useful to have the patients divided according to their class of diet: (*a*) Milk or simple diets, (*b*) ordinary. But if this makes difficulties it would be wiser to confine the record simply to numbers. The housekeeping sister should post it up daily, and it will form a valuable record for estimating expenditure. All extra nurses engaged and extra persons of any description will thus take their place as consumers of food, and even casual visitors will count. Moreover, the holidays of the staff will be shown, and, in fact, every variation in number which is experienced right through the establishment.

### The Importance of Method.

The next step towards getting a firm grip on the reins is to ascertain precisely how much the patients cost. We noted with pleasure the criticism of "Another Isolation Matron" in regard to paper estimates of the cost of diet. It seems so easy to calculate how much a given number of persons will cost when the exact weight and measure of their food and drink is rigidly laid down, with the price of the articles consumed. But if this were all, housekeeping would not be the complicated art which it is found to be in institutions. Even in well-managed households it may safely be calculated that at least one extra person in twenty is boarded, the food, it should be understood, not being consumed by any one but wasted *en passant*. Thus, when these paper calculations are made, a margin of at least 5 per cent. should be allowed for over and above the actual consumers of the food. When the management is lax these invisible mouths consume an incredible amount, and in order to ascertain to what extent leakage is going on there is no way but to keep the patients' food supplies rigidly separate from those of the other members of the household, and check the cost by the numbers entered in the housekeeper's book.

We have met very clever hospital housekeepers who do not believe that any purpose is served by separating the food supplies of patients from those of the household. We have not been convinced by their arguments, but we have reached the conclusion that in the vast majority of institutions the custom of using all the supplies in common is so firmly rooted that there is little to be gained by arguing. This bad custom is largely to be accounted for by shortage of labour and of time for any clerical work.

### Some Vital Principles.

However this may be, it is indisputable that the housekeeper must know how much it actually costs to feed the patients before she can adequately control the cost of boarding the rest of the household. To this end, whatever the general custom be, she ought to make a definite effort, at any rate for one week, to keep all the patients' supplies separate. One table or part of the table in the kitchen should be kept for food intended solely for their consumption, and a slate should be ruled on which the cook should enter particulars of all that is used for them at every meal. The calculation is facilitated by the small number of articles used in patients' diets. Milk for their use can easily be kept separate if the precaution be taken of sending up additional milk in separate vessels for use in the ward kitchens for nurses' teas, etc. For one week, at any rate, the patients' joints can be used only for them. For one week the cook can be troubled to weigh off the amount of potatoes necessary, and weigh (not estimate) the amount of rice she uses for their puddings, the amount of Benger's Food, or other extras required for the diets. For one week the stock-pot for the patients' broth can be used for this purpose and for no other, and their loaves counted.

At the end of the week the cost of what has been consumed solely by the patients must be computed, not a difficult task, since all the articles of which it is composed are standardised in price. This amount is to be divided by the average daily number of patients actually in the wards during this week, and the daily cost of the patient will then have been arrived at.

It may be said that the experience of one week is of small value. Its value lies in the use which is made of it. It can, for instance, be taken as a standard of comparison with those paper statistics justly suspect of our correspondent. But to reach its full value it should be checked from time to time by the same process; if this be repeated, say, once a quarter or a few days after a change of cook or housekeeper, the daily cost of the patient becomes at length a matter of certainty, within a farthing or two. When this has been accomplished, the foundation for good housekeeping has been laid.

